# An Improved Large Signal Model for 0.1 μm AlGaN/GaN High Electron Mobility Transistors (HEMTs) Process and Its Applications in Practical Monolithic Microwave Integrated Circuit (MMIC) Design in W band

**DOI:** 10.3390/mi9080396

**Published:** 2018-08-10

**Authors:** Junfeng Li, Shuman Mao, Yuehang Xu, Xiaodong Zhao, Weibo Wang, Fangjing Guo, Qingfeng Zhang, Yunqiu Wu, Bing Zhang, Tangsheng Chen, Bo Yan, Ruimin Xu, Yanrong Li

**Affiliations:** 1 School of Electronic Science and Engineering (National Exemplary School of Microelectronics), University of Electronic Science and Technology of China, Chengdu 611731, China; lijunfeng_EE@163.com (J.L.); maoshuman@163.com (S.M.); zhaoxiaodong@std.uestc.edu.cn (X.Z.); a2277462594@163.com (Q.Z.); yqwu@uestc.edu.cn (Y.W.); yanbo@ee.uestc.edu.cn (B.Y.); rmxu@uestc.edu.cn (R.X.); yrli@uestc.edu.cn (Y.L.); 2 Nanjing Electronic Devices Institute, Nanjing 210016, China; bobommic@163.com (W.W.); fjiguo@163.com (F.G.); binzhang_cetc55@aliyun.com (B.Z.); chentsh@vip.sina.com (T.C.)

**Keywords:** AlGaN/GaN HEMT, DIBL effect, channel length modulation, power amplifier, W band

## Abstract

An improved empirical large signal model for 0.1 µm AlGaN/GaN high electron mobility transistor (HEMT) process is proposed in this paper. The short channel effect including the drain induced barrier lowering (DIBL) effect and channel length modulation has been considered for the accurate description of DC characteristics. In-house AlGaN/GaN HEMTs with a gate-length of 0.1 μm and different dimensions have been employed to validate the accuracy of the large signal model. Good agreement has been achieved between the simulated and measured S parameters, I-V characteristics and large signal performance at 28 GHz. Furthermore, a monolithic microwave integrated circuit (MMIC) power amplifier from 92 GHz to 96 GHz has been designed for validation of the proposed model. Results show that the improved large signal model can be used up to W band.

## 1. Introduction

Wide band gap semiconductor Gallium Nitride (GaN) high electron mobility transistors (HEMTs) are excellent candidates in high frequency power electronics due to their unique advantages of higher breakdown voltage and higher output power density [1]. With the rapid development of process, the feature size of GaN HEMTs have been shrinking to less than 0.1 µm. GaN HEMTs with good performance for application in W band have been reported [2,3,4,5]. Also, over the past few years, several GaN HEMT based monolithic microwave integrated circuits (MMICs) up to W-band have been developed, due to their applications in high speed wireless communications or radar systems [6]. A GaN MMIC power amplifier at 91 GHz was reported to have 1.7 W output power that is associated with 11% power added efficiency [7]. A W-Band MMIC power amplifier with 3.46 W/mm output power density and 21% associated power added efficiency was then reported. The associated power gain is 13.7 dB. It offers a peak small signal gain of 16.7 dB over 90–97 GHz [2].

For applications of these devices in circuit design, compact nonlinear device modeling plays an important role in practical design. Recently, a few physical based compact models have sprung up due to their advantages in less fitting parameters and good accuracy up to the Ka band [8,9,10,11]. However, things will be different when the frequency is up to W band. Firstly, the parasitic effect will become obvious with the increasing of frequency and make the parameter extraction more difficult [12,13]. This problem can be solved by FW-EM (Full-wave electromagnetic) simulation [14]. Secondly, along with the reduction of feature size, the short channel effect becomes obvious. This phenomenon will in the end give rise to shift of threshold voltage. Thirdly, the gradual channel approximation (GCA) that is used in many kinds of physical based compact model [15,16] is no more effective as the channel length modulation is obvious in short channel devices. These effects will largely decrease the accuracy of physical based compact model. The empirical modeling method has been widely used due to their excellent performance in convergence and accuracy [17,18,19,20,21,22]. An effective validation of large signal model is validated by on-wafer load-pull measurement [23,24]. However, due to the complication of load-pull measurement, only one input/output impedance is validated. Nevertheless, more input/output impedances need to be validated for a large signal model in practical MMIC power amplifier design [25].

In this paper, the short channel effect, including the DIBL effect and channel length modulation, is studied. An improvement for the accuracy of the area near the pinch-off region in IV curve is performed based on an empirical modeling method as the GCA is no more effective in most physical based model. In-house AlGaN/GaN HEMTs with gate length of 0.1 μm is used for validation of the model. Performance, including S parameters, DC characteristics, and large signal characteristics at 28 GHz is validated by on-wafer measurement. Finally, a MMIC power amplifier is designed based on the proposed model for further validation.

This paper is organized as follows. In Section 2, the investigation on short channel effect is presented. The modeling method of it, which is based on an empirical method, is given in detail. In Section 3, the proposed large signal model is validated with two GaN HEMTs with different gate width. In Section 4, a MMIC power amplifier based on the large signal model in this work is designed for further validation of the model in W band. Finally, in Section 5, the conclusion of this work is presented.

## 2. Model Description

### 2.1. Short Channel Effects

**Figure 1:**
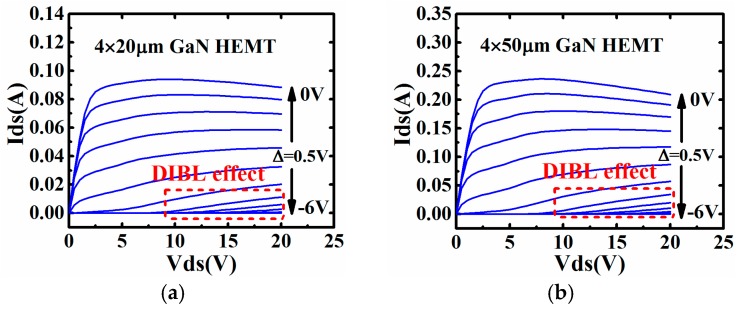
Drain induced barrier lowering (DIBL) effect in Static IV curves of 0.1 μm AlGaN/GaN high electron mobility transistor (HEMT) with different gate width: (**a**) 4 × 20 μm and (**b**) 4 × 50 μm.

Along with the decrease of gate length, the short channel effect, such as the drain induced barrier lowering (DIBL) effect will become obvious. The thickness of the barrier will not only be modulated by gate voltage, but also drain voltage. This will, in the end, lead to the drift of threshold voltage along with the drain voltage. This phenomenon can be easily captured in the static IV curve of 0.1 μm AlGaN/GaN HEMTs with different gate width in this work, which have been shown in Figure 1.

It can be seen from Figure 1 that the DIBL effect will weaken the effect that is brought by gate voltage. The device will be turned from off-state to on-state with the rise of drain voltage. This phenomenon must be taken into consideration, especially for high efficiency power amplifier or switching applications.

In order to accurately describe the output performance of AlGaN/GaN HEMTs with short gate length in large signal modeling, the short channel effect, including the DIBL effect and channel length modulation, should be taken into consideration. An empirical method that is based on the Angelov model is employed for the devices in this work. As we know that the coefficients of the *ψ* polynomial in Angelov model, which is shown in Equation (1), mainly affect the accuracy of the region close to pinch-off state.

(1) $$\psi =P_{1}\times (V_{gs}-V_{pk1})+P_{2}\times {\left(V_{gs}-V_{pk2}\right)}^{2}+P_{3}\times {\left(V_{gs}-V_{pk3}\right)}^{3}$$

where *V_gs_* refers to the gate-source voltage. *V_pkn_* (*n* = 1, 2, 3) are fitting parameters. *P_n_* (*n* = 1, 2, 3) are fitting coefficients of the *ψ* polynomial.

To accurately model the DIBL effect, the drain-source voltage *V_ds_* has been included in *P_n_* (*n* = 1, 2, 3) to take the modulation effect of *V_ds_* into consideration, as shown in Equation (2).

(2) $$P_{n}=P_{n0}+(P_{n1}\times V_{ds}-P_{n0})\times \tanh\left(\alpha P_{n2}\times V_{ds}\right)\;\left(n=1,2,3\right)$$

where *P_n_*_0_, *P_n_*_1_, *P_n_*_2_ and *α* are all fitting parameters.

**Figure 2:**
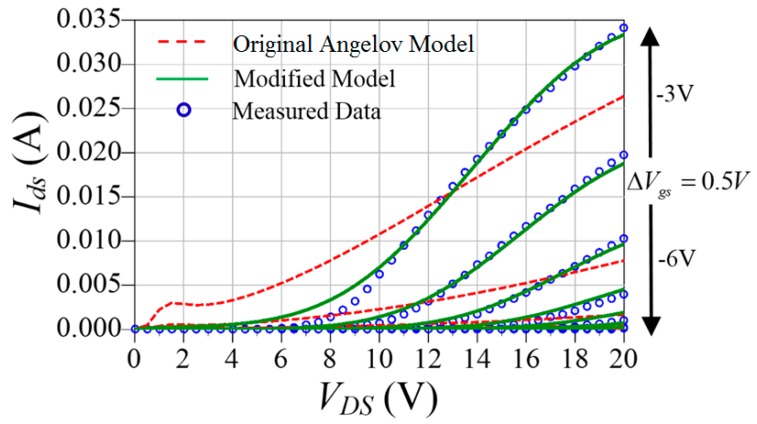
Comparison between simulated and measured results when *V_gs_* is close to pinch-off voltage.

The modification was validated by a comparison between simulation results and measured data. The comparison between the original Angelov model and modified one are shown in Figure 2. The gate-source voltage *V_gs_* is from −6 V to −3 V and the drain source voltage *V_ds_* is from 0 V to 20 V.

It is clear in Figure 2 that the original Angelov model cannot accurately describe the DC characteristics when *V_gs_* is close to the pinch-off voltage. The DIBL effect can be successfully modeled by using proposed model.

**Figure 3:**
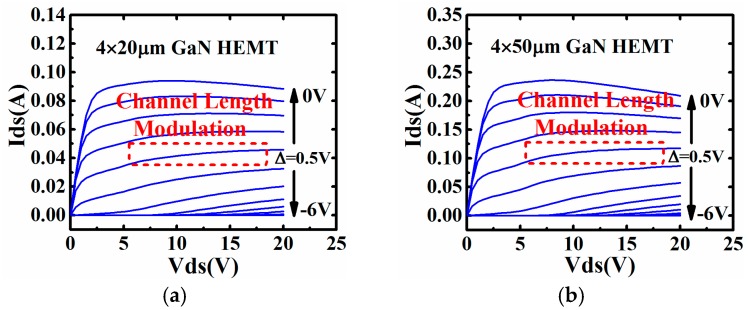
Channel length modulation effect in Static IV curves of 0.1 μm AlGaN/GaN HEMT with different gate width: (**a**) 4 × 20 μm and (**b**) 4 × 50 μm.

Apart from the DIBL effect, the channel length modulation can also be captured in the static IV curves, as shown in Figure 3.

**Figure 4:**
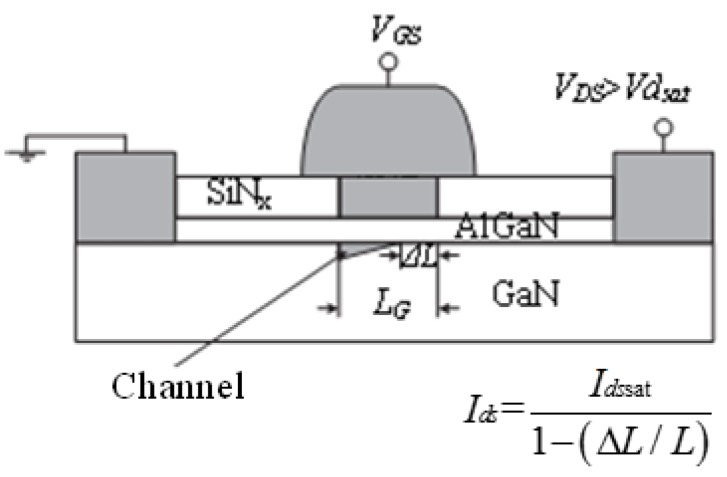
The schematic diagram of the short channel modulation effect.

It clearly shows that the partial derivative of *I_ds_* to *V_ds_* is not equal to zero due to channel length modulation. The channel length effect is mainly induced by expanding of the depletion region towards the source. The effective channel is then shortened. This phenomenon is shown in Figure 4.

### 2.2. Large Signal Model up to W Band 

**Figure 5:**
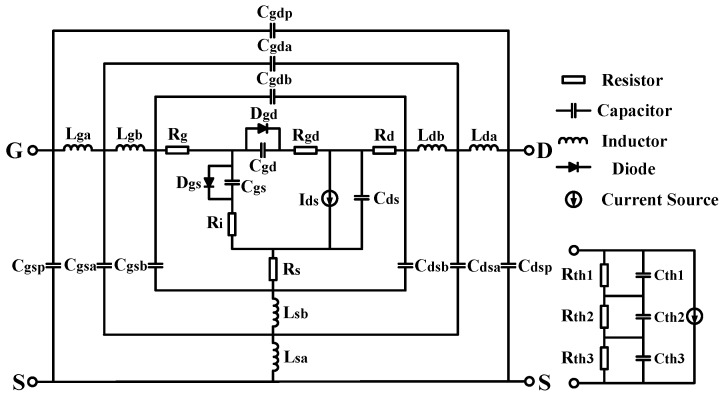
Topology of Large signal model up to W band.

With the frequency up to W band, the RF dispersion will become more and more obvious due to the parasitic effects inside devices. A wide band small signal model [14], which has been proved to be able to cover the frequency band from 0.2–110 GHz, is employed in this work. The topology of the large signal model is shown in Figure 5.

The main part of the nonlinear current model as well as the capacitance model, including *C_gs_* and *C_gd_* mentioned in [21], is employed in this work. The improvement for accurate characterization of short channel effect, which is mentioned in the previous section, has also been included in the nonlinear current model. In order to accurately characterize the self-heating effect in AlGaN/GaN HEMT. The three-pole thermal network in [25] is used. Thermal resistances as well as the thermal capacitances are extracted by a method based on FEM simulation in ANSYS. The trapping effect is modeled by the equivalent voltage method in [26]. The scalability of the model parameters, including the *I_pk_*_0_, *R_th_,* and *C_th_* has been realized with the method that is mentioned in [22] for practical monolithic microwave integrated circuit design. With the help of MATLAB coding, model parameters, except the coefficients in Equation (2), are all extracted with the method in [27]. In terms of parameters in Equation (2), they are all extracted by fitting the transfer characteristics curve with the least square method.

## 3. Model Validation 

### 3.1. Small Signal Characterization

**Figure 6:**
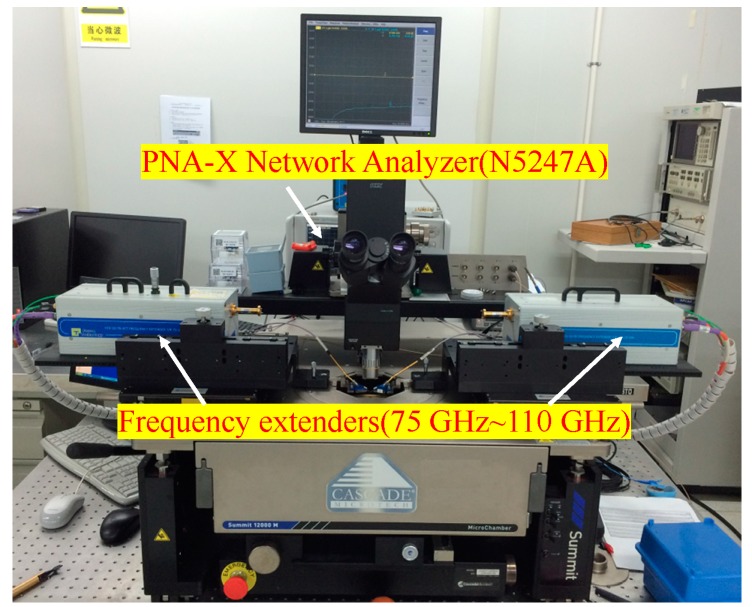
On-wafer measurement system for small signal characteristics.

The large signal model was embedded into Keysight ADS (Advanced Design System) by a symbolically defined device (SDD) tool. Small signal characteristics of the devices are measured by cascade probe station (Summit 11000B, FormFactor, Livermore, CA, USA), which is shown in Figure 6. The vector network analyzer is Keysight N5247A (Keysight Technologies, Santa Rosa, CA, USA). The frequency extenders close to probes are used to achieve the S parameters ranging from 75 GHz to 110 GHz as the vector network analyzer can only reach up to 67 GHz.

**Figure 7:**
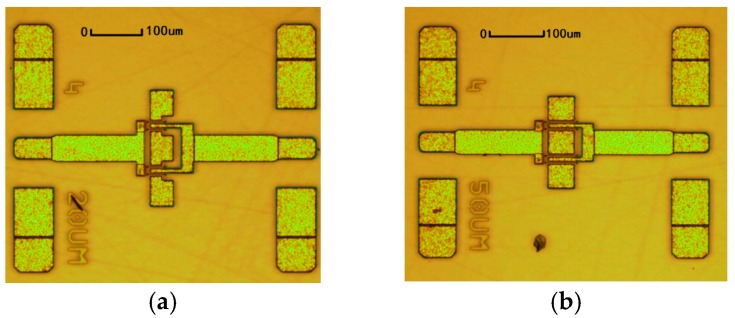
0.1 μm AlGaN/GaN HEMTs: (**a**) 4 × 20 μm and (**b**) 4 × 50 μm.

The proposed model was validated by 0.1 μm AlGaN/GaN HEMTs with different gate width. AlGaN/GaN HEMTs were all fabricated on a 4-inch SiC substrate. T-shape-gate technology was introduced to reduce the contact resistance. The *f_T_* of the 0.1 μm GaN process is 90 GHz, while *f_max_* is 220 GHz. The peak power density for a specific device can reach up to 3.46 W/mm. The photography of devices is shown in Figure 7.

**Figure 8:**
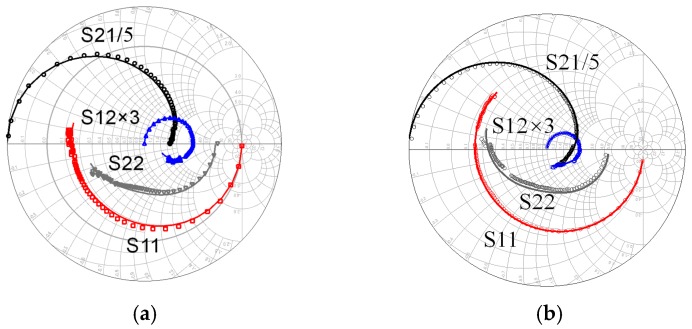
Comparison of simulated and measured S-parameters: (**a**) 4 × 20 μm at *Vgs* = −2 V, *Vds* = 10 V and (**b**) 4 × 50 μm at *Vgs* = −1 V, *Vds* = 15 V.

The comparison of simulated and measured S parameters is shown in Figure 8. Results show that the proposed model can predict the small signal characteristics ranging from 0.2 GHz to 110 GHz for devices with different gate width and under different bias.

### 3.2. The Large Signal Model Validation

**Figure 9:**
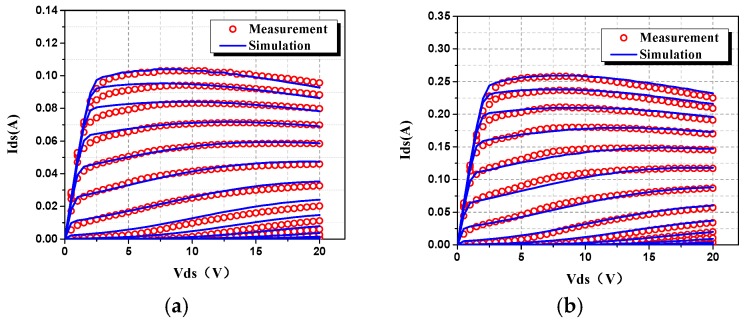
Comparison of simulated and measured DC characteristics of 0.1 μm AlGaN/GaN HEMTs: (**a**) 4 × 20 μm and (**b**) 4 × 50 μm.

The DC characteristics for the proposed scalable large signal model was validated by different gate width, including 4 × 20 μm and 4 × 50 μm, as shown in Figure 9. The gate-source voltage *V_gs_* is investigated from −6 V to 0 V, while the drain-source voltage *V_ds_* is from 0 V to 20 V for these two devices.

Figure 9 shows that the DIBL effect is accurately characterized based on the improvement in Equation (2). The channel length modulation effect is also the same.

**Figure 10:**
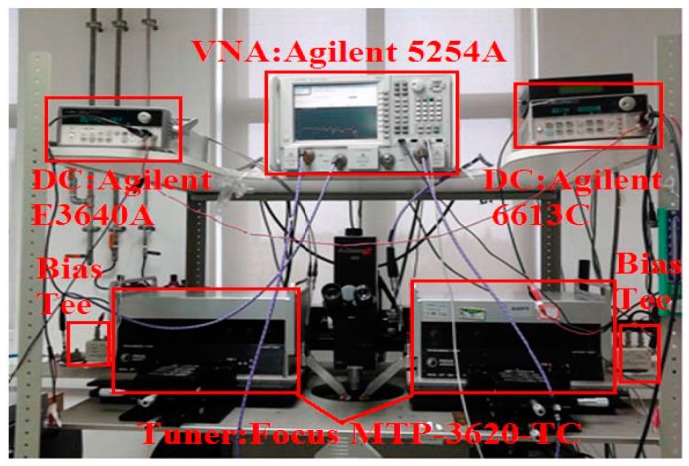
Photograph of on-wafer load–pull system setup.

Due to the absent of W band load-pull system, the load pull performance at 28 GHz was used to validate the large signal model first, as shown in Figure 10. The system is on cascade probe station (Summit 12000, FormFactor, Livermore, CA, USA), the input signal generator is Agilent E8257D (Keysight Technologies, Santa Rosa, CA, USA), and the output power is detected by power meter Agilent N1912A (Keysight Technologies, Santa Rosa, CA, USA) and Vector Network Analyzer (Keysight Technologies, Santa Rosa, CA, USA).

**Figure 11:**
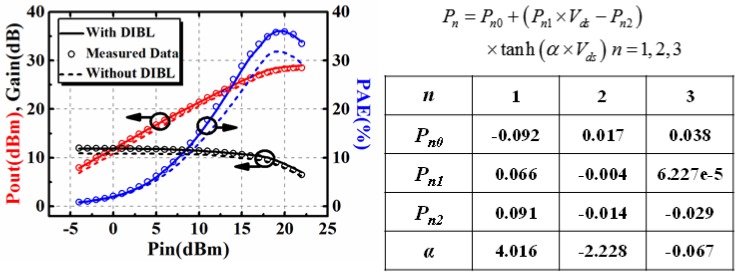
Investigation on the influence brought by DIBL effect on large signal performance.

The maximum output power load-pull measurement is performed. The bias is chosen at *V_gs_* = −2.6 V, *V_ds_* = 15 V, which is at deep class AB working state. The quiescent drain current is 82 mA at this bias. The optimum source and load resistance for the maximum output power are *Z_S_* = (13.44 + 12.41 × j) Ω and *Z_L_* = (27.19 + 27.44 × j) Ω. The power sweep was then performed based on the optimum resistance with the input power ranging from −4 dBm to 22 dBm. The comparison between the simulated and measured results, including output power (Pout), gain, and power added efficiency (PAE) are shown in Figure 11. Also, the influence that is brought by the DIBL effect has also been investigated in Figure 11. Results show that the DIBL effect will lead to the reduction of Pout, gain, and PAE. This can be explained by the variation of static bias point due to the DIBL effect.

**Figure 12:**
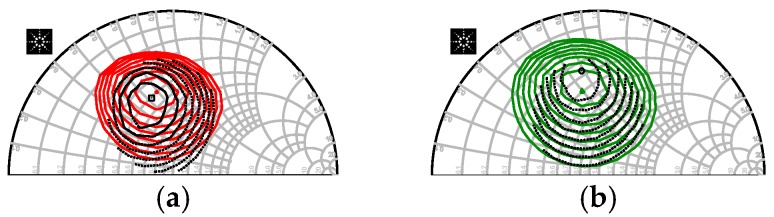
Comparison between simulated impedance chart and measured one: (**a**) maximum Pout and (**b**) maximum power added efficiency (PAE).

The simulated and measured impedance charts achieved by maximum Pout and PAE load-pull measurement are presented in Figure 12.

## 4. W Band MMIC Power Amplifier Design

**Figure 13:**
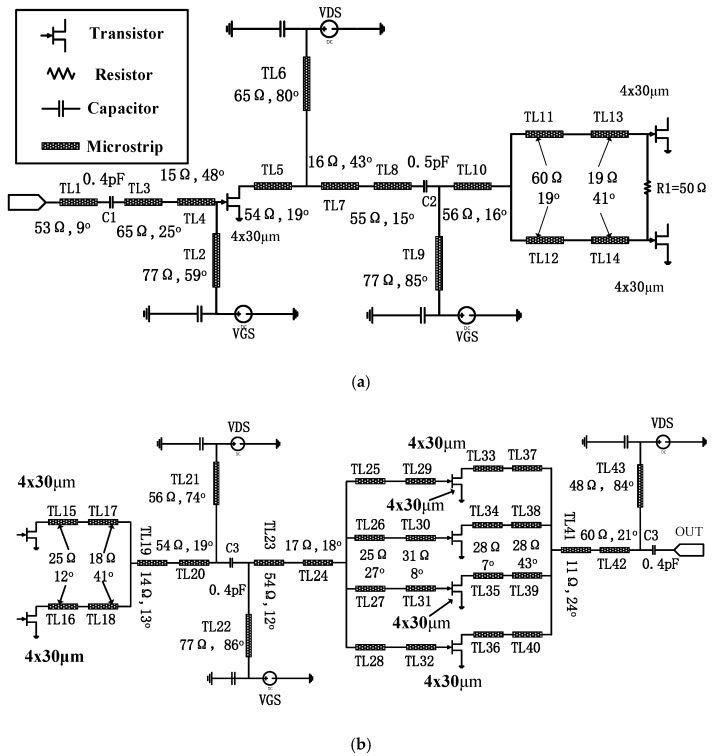
Schematic of W band amplifier: (**a**) Preceding stage and (**b**) Post stage.

For further validation of the proposed large signal model for applications in the W band, a MMIC power amplifier whose operation frequency is 92 GHz–96 GHz was designed. Based on the above large signal model, a W-band power amplifier is designed. Figure 13 presents the schematic of the W band amplifier.

**Figure 14:**
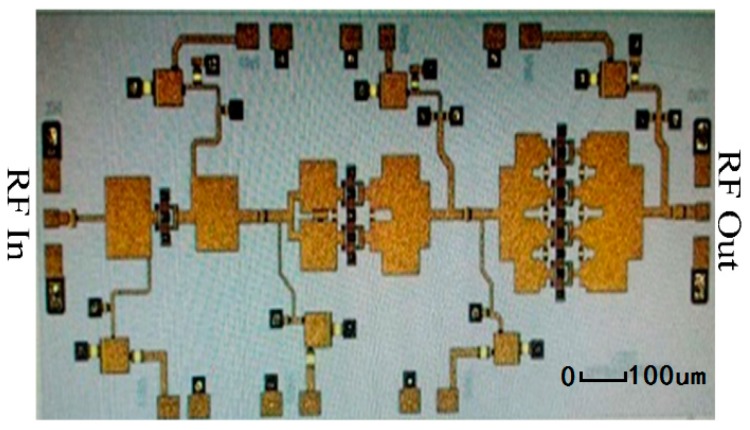
Photograph of a W-band Gallium Nitride (GaN) monolithic microwave integrated circuits (MMIC) amplifer.

The output stage used the planar spatial power combiner to realize the impedance transformation and combine the four-way power element. The millimeter wave GaN device is very easy to oscillation at low frequency due to the high gain. Multi-order RC network was used to improve the stability of the circuit. In order to enable the former stage to have enough power to drive the latter stage, the driving ratio of amplifier circuit is 1:2:4. Passive components include micro-strip line, MIM (Metal-insulator-Metal) capacitance, and resistor. All of the passive components were simulated by EM simulator in ADS. Figure 14 shows photograph of a W-band GaN MMIC amplifier.

**Figure 15:**
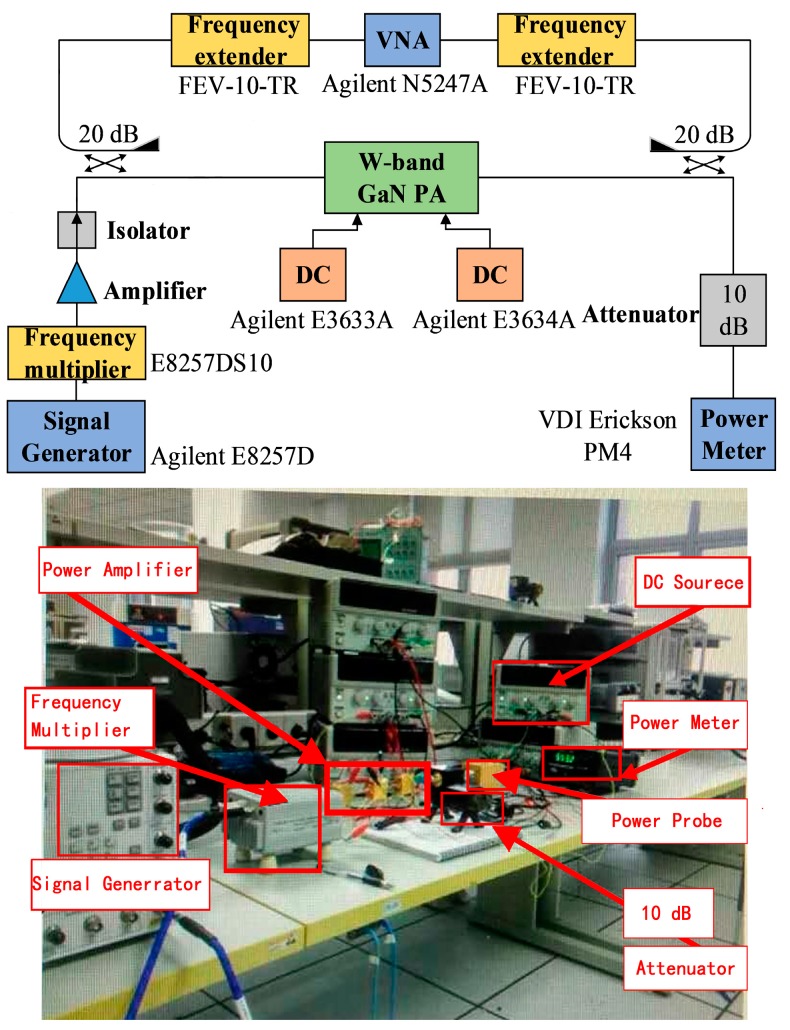
Photograph of the measurement setup for the W band MMIC power amplifier.

The chip was loaded into a jig for measurement. The schematic of the measurement setup for large-signal measurements is shown in Figure 15. The large signal measurement was performed at room temperature. The commercial amplifier, frequency multiplier, and signal analyzer in Figure 15 are used to assistant the measurement. Other instruments including power meter (VDI Erickson, Virginia Diodes, Inc., Charlottesville, VA, USA), DC sources (Agilent E3633A and E3634A, Keysight Technologies, Santa Rosa, CA, USA), and attenuator (Rebes, Suzhou, China) were also employed. The amplifier is measured in CW (Continuous Wave) mode over 90 GHz–97 GHz frequency. The device was bias at *V_ds_* = 15 V and *V_gs_* = −2 V.

**Figure 16:**
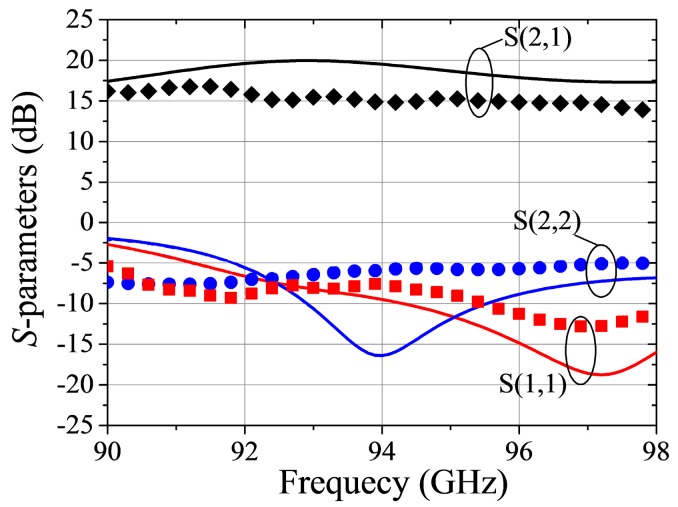
Measured (solid) and simulated (Symbol) S parameters of W band MMIC amplifier.

**Figure 17:**
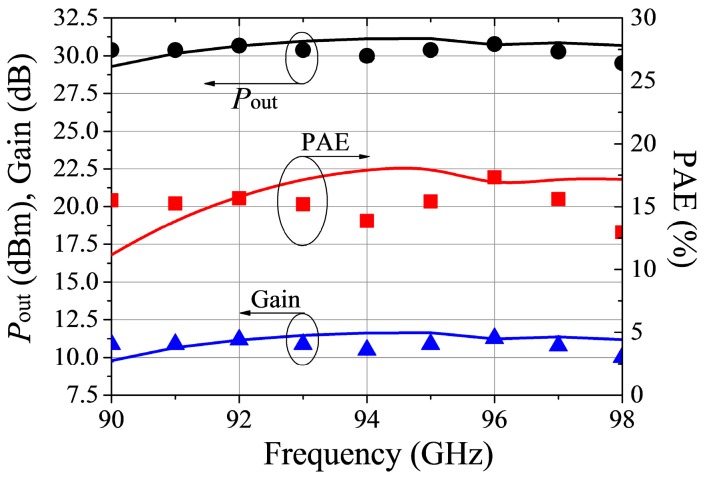
Measured (Symbol) and simulated (solid) large-signal characteristics of the W-band MMIC PA.

Figure 16 displays measured and simulated S-parameters of the MMIC amplifier. The difference in Figure 16 may come from the cavity and gold wire used for assisting the measurement. Their influence on frequency shift has not been taken into consideration during the MMIC design. However, this accuracy is sufficient for the application of practical circuit design. Figure 17 shows Gain, PAE, and output power. Over 90 GHz–97 GHz frequency range, the output power is greater than 1 W. The peak output power is 1.2 W. Except for 94 GHz and 98 GHz, the measured PAE was greater than 15%. 

## 5. Conclusions

In this paper, an improved large signal model for AlGaN/GaN HEMT up to the W band is presented. The short channel effects including the DIBL effect and channel length modulation are added in the Angelov model. In-house AlGaN/GaN HEMTs with gate length of 0.1 μm are used for the validation of the model. A MMIC power amplifier is designed based on the proposed model for further validation. Results show that the large signal model can give good accuracy up to W band. The results of this paper can provide guidance to many other kinds FET (Field Effect Transistor) devices modeling in the W band. Also, they are useful for the improvement of the GaN process and also are helpful for the practical MMIC design in the W band.

## References

[1] Mishra U.K., Shen L., Kazior T.E., Wu Y.F. (2008). Gan-based RF power devices and amplifiers. Proc. IEEE..

[2] Shaobing W., Jianfeng G., Weibo W., Junyun Z. (2016). W-band MMIC PA with ultrahigh power density in 100-nm AlGaN/GaN technology. IEEE Trans. Electron Devices.

[3] Wienecke S., Romanczyk B., Guidry M., Li H., Ahmadi E., Hestroffer K., Zheng X., Keller S., Mishra U.K. (2017). N-polar gan cap mishemt with record power density exceeding 6.5 W/mm at 94 GHz. IEEE Electron Device Lett..

[4] Xing W., Liu Z., Ranjan K., Ng G.I., Palacios T. (2018). Planar nanostrip-channel Al_2_O_3_/InAIN/GaN MISHEMTs on Si with improved linearity. IEEE Electron Device Lett..

[5] Romanczyk B., Wienecke S., Guidry M., Li H., Ahmadi E., Zheng X., Keller S., Mishra U.K. (2018). Demonstration of constant 8 W/mm power density at 10, 30, and 94 GHz in state-of-the-art millimeter-wave N-polar GaN MISHEMTs. IEEE Trans. Electron Devices.

[6] Niida Y., Kamada Y., Ohki T., Ozaki S., Makiyama K., Minoura Y., Okamoto N., Sato M., Joshin K., Watanabe K. 3.6 W/mm high power density W-band InAlGaN/GaN HEMT MMIC power amplifier. Proceedings of the 2016 IEEE Topical Conference on Power Amplifiers for Wireless and Radio Applications (PAWR).

[7] Brown A., Brown K., Chen J., Hwang K.C., Kolias N., Scott R. W-band GaN power amplifier MMICs. Proceedings of the 2011 IEEE MTT-S International Microwave Symposium.

[8] Cheng X., Wang Y. (2011). A surface-potential-based compact model for AlGaN/GaN MODFETs. IEEE Trans. Electron Devices.

[9] Khandelwal S., Chauhan Y.S., Fjeldly T.A. (2012). Analytical modeling of surface-potential and intrinsic charges in AlGaN/GaN HEMT devices. IEEE Trans. Electron Devices.

[10] Deng W., Huang J., Ma X., Liou J.J. (2015). An explicit surface potential calculation and compact current model for AlGaN/GaN HEMTs. IEEE Electron Device Lett..

[11] Radhakrishna U., Choi P., Grajal J., Peh L.S., Palacios T., Antoniadis D. Study of RF-circuit linearity performance of GaN HEMT technology using the MVSG compact device model. Proceedings of the 2016 IEEE International Electron Devices Meeting (IEDM).

[12] Dambrine G., Cappy A., Heliodore F., Playez E. (1988). A new method for determining the FET small-signal equivalent circuit. IEEE Trans. Microw. Theory Tech..

[13] Jarndal A., Kompa G. (2005). A new small-signal modeling approach applied to GaN devices. IEEE Trans. Microw. Theory Tech..

[14] Jia Y., Xu Y., Xu R., Li Y. (2018). An accurate parasitic parameters extraction method based on FW-EM for AlGaN/GaN HEMT up to 110 GHz. Int. J. Numer. Model. Electron. Netw. Devices Fields.

[15] Wen Z., Xu Y., Chen Y., Tao H., Ren C., Lu H., Wang Z., Zheng W., Zhang B., Chen T. (2017). A quasi-physical compact large-signal model for AlGaN/GaN HEMTs. IEEE Trans. Microw. Theory Tech..

[16] Wu Q., Xu Y., Chen Y., Wang Y., Fu W., Yan B., Xu R. (2018). A scalable multiharmonic surface-potential model of AlGaN/GaN HEMTs. IEEE Trans. Microw. Theory Tech..

[17] Crupi G., Xiao D., Schreurs D.M.M.P., Limiti E., Caddemi A., Raedt W.D., Germain M. (2006). Accurate multibias equivalent-circuit extraction for GaN HEMTs. IEEE Trans. Microw.Theory Tech..

[18] Jardel O., Groote F.D., Reveyrand T., Jacquet J.C., Charbonniaud C., Teyssier J.P., Floriot D., Quere R. (2007). An electrothermal model for AlGaN/GaN power HEMTs including trapping effects to improve large-signal simulation results on high VSWR. IEEE Trans. Microw. Theory Tech..

[19] Liu L.S., Ma J.G., Ng G.I. (2009). Electrothermal large-signal model of III–V FETs including frequency dispersion and charge conservation. IEEE Trans. Microw. Theory Tech..

[20] Zhao X., Xu Y., Jia Y., Wu Y., Xu R., Li J., Hu Z., Wu H., Dai W., Cai S. (2017). Temperature-dependent access resistances in large-signal modeling of millimeter-wave AlGaN/GaN HEMTs. IEEE Trans. Microw. Theory Tech..

[21] Wang C., Xu Y., Yu X., Ren C., Wang Z., Lu H., Chen T., Zhang B., Xu R. (2014). An electrothermal model for empirical large-signal modeling of AlGaN/GaN HEMTs including self-heating and ambient temperature effects. IEEE Trans. Microw. Theory Tech..

[22] Xu Y., Wang C., Sun H., Wen Z., Wu Y., Xu R., Yu X., Ren C., Wang Z., Zhang B. (2017). A scalable large-signal multiharmonic model of AlGaN/GaN HEMTs and its application in C-band high power amplifier MMIC. IEEE Trans. Microw. Theory Tech..

[23] Joshin K., Ozaki S., Ohki T., Okamoto N., Niida Y., Makiyama K. Millimeter-wave GaN HEMT model with VDS dependence of CDS for power amplifier applications. Proceedings of the 2014 Asia-Pacific Microwave Conference.

[24] Cutivet A., Altuntas P., Defrance N., Okada E., Avramovic V., Lesecq M., Hoel V., Jaeger J.C.D., Boone F., Maher H. Large-signal modeling up to W-band of AlGaN/GaN based high-electron-mobility transistors. Proceedings of the 2015 10th European Microwave Integrated Circuits Conference (EuMIC).

[25] King J.B., Brazil T.J. (2013). Nonlinear electrothermal GaN HEMT model applied to high-efficiency power amplifier design. IEEE Trans. Microw. Theory Tech..

[26] Yuk K.S., Branner G.R., McQuate D.J. (2009). A wideband multiharmonic empirical large-signal model for high-power GaN HEMTs with self-heating and charge-trapping effects. IEEE Trans. Microw. Theory Tech..

[27] Wen Z., Xu Y., Wang C., Zhao X., Chen Z., Xu R. (2017). A parameter extraction method for GaN HEMT empirical large-signal model including self-heating and trapping effects. Int. J. Numer. Model. Electron. Netw. Devices Fields.

